# Data showing effects of a PI3K-δ inhibitor on neutrophil superoxide production during FPR2 activation and reactivation

**DOI:** 10.1016/j.dib.2020.106185

**Published:** 2020-08-17

**Authors:** André Holdfeldt, Martina Sundqvist, Claes Dahlgren, Huamei Forsman

**Affiliations:** Department of Rheumatology and Inflammation Research, Institute of Medicine, Sahlgrenska Academy, University of Gothenburg, Guldhedsgatan 10A, Gothenburg 413 46, Sweden

**Keywords:** Neutrophils, Reactive oxygen species, PI3K, FPR2, PAFR, Receptor cross-talk

## Abstract

Reactive oxygen species (ROS) generated by the NADPH oxidase are crucial for antimicrobial host defense and also play a role in the regulation of inflammatory processes. Signals generated by formyl peptide receptor 2 (FPR2) activate the neutrophil ROS generating NADPH oxidase; such signals are mediated when the receptors bind an activating agonist, as well as when agonist desensitized FPR2 are reactivated by the receptor for platelet-activating factor (PAF). We present data on the effects of Idelalisib, a specific inhibitor for the PI3Kδ isoform, on ROS production during FPR2 activation and reactivation by PAF, respectively. Neutrophils were isolated from peripheral blood of healthy adults obtained from the blood bank at Sahlgrenska University Hospital and ROS release was measured using isoluminol-amplified chemiluminescence.

**Table:** 

**Subject**	*Cell biology and immunology*
**Specific subject area**	*G protein-coupled receptor signaling, PI3K-δ, formyl peptide receptor 2, platelet-activating factor receptor, human neutrophils, inflammation*
**Type of data**	*Figures and graphs.*
**How data were acquired**	*Data were obtained from in vitro experiments with human neutrophils using a luminometer (Biolumat LB 9505 (Berthold Co, Wildbad, Germany) and an isoluminol-amplified chemiluminescence assay system to monitor superoxide production*.
**Data format**	*Raw data analyzed and processed*
**Parameters for data collection**	*Human blood neutrophils isolated from buffy coats were activated with receptor agonists in the presence or absence of a PI3K-δ specific inhibitor and receptor downstream function was monitored as production of superoxide anions.*
**Description of data** **collection**	*Production of superoxide anions by the neutrophil NADPH-oxidase was measured by an isoluminol-enhanced chemiluminescence system which records light emission continuously over time. To determine the impact of the specific PI3K-δ specific inhibitor on receptor agonists induced NADPH-oxidase activity the relative light emission/superoxide production (counts per minute; CPM) was analyzed and compared.*
**Data source location**	*Department of Rheumatology and Inflammation Research, Institute of Medicine, Sahlgrenska Academy, Gothenburg University* *Gothenburg, Sweden*
**Data accessibility**	*Data are hosted by the article* *Data files from Biolumat LB9505 are available by request to andre.holdfeldt@rheuma.gu.se*

**Value of the Data**

• These data are the first to show the involvement of PI3K-δ in FPR2 mediated activation of the neutrophils NADPH-oxidase, an electron transport system that is essential for microbial killing and the regulation of inflammatory processes.
• These results expand the current knowledge about GPCR signaling which may benefit research of several inflammatory diseases including the activating PI3K-delta syndrome (APDS) as well as research focused on the role of FPR2 as a regulator of inflammation.
• The findings can be used to further characterize the role of PI3K-δ in inflammatory settings, by additional studies of the functions of PI3K-δ in regulating GPCR signaling and oxidative burst activity in inflammatory diseases.

## 1 Data description

### 1.1 Data

**Fig 1:**
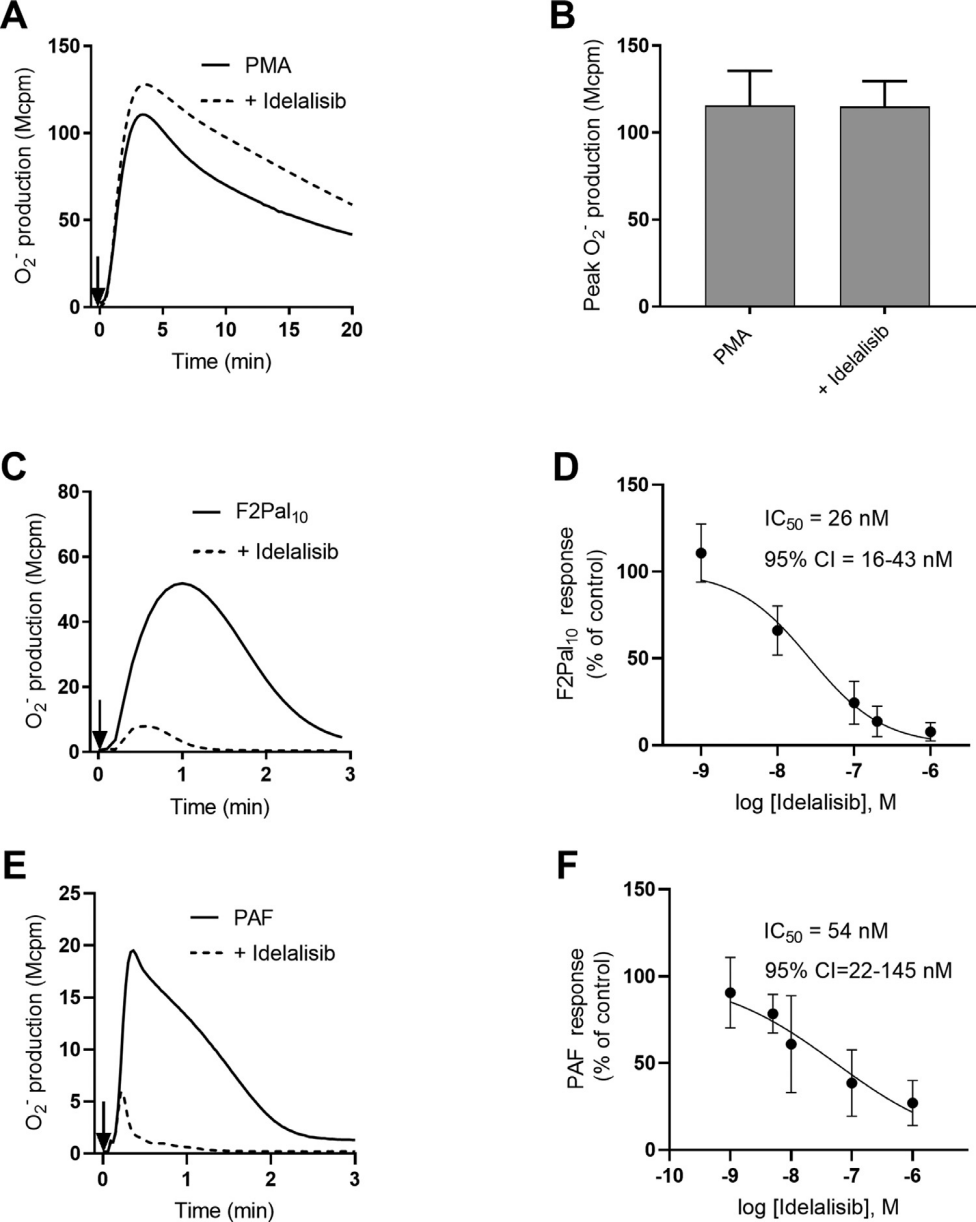
Effect of the PI3K-δ isoform specific inhibitor Idelalisib on neutrophil O_2_^−^ production induced by PMA, F2Pal_10_ or PAF.

**Fig 2:**
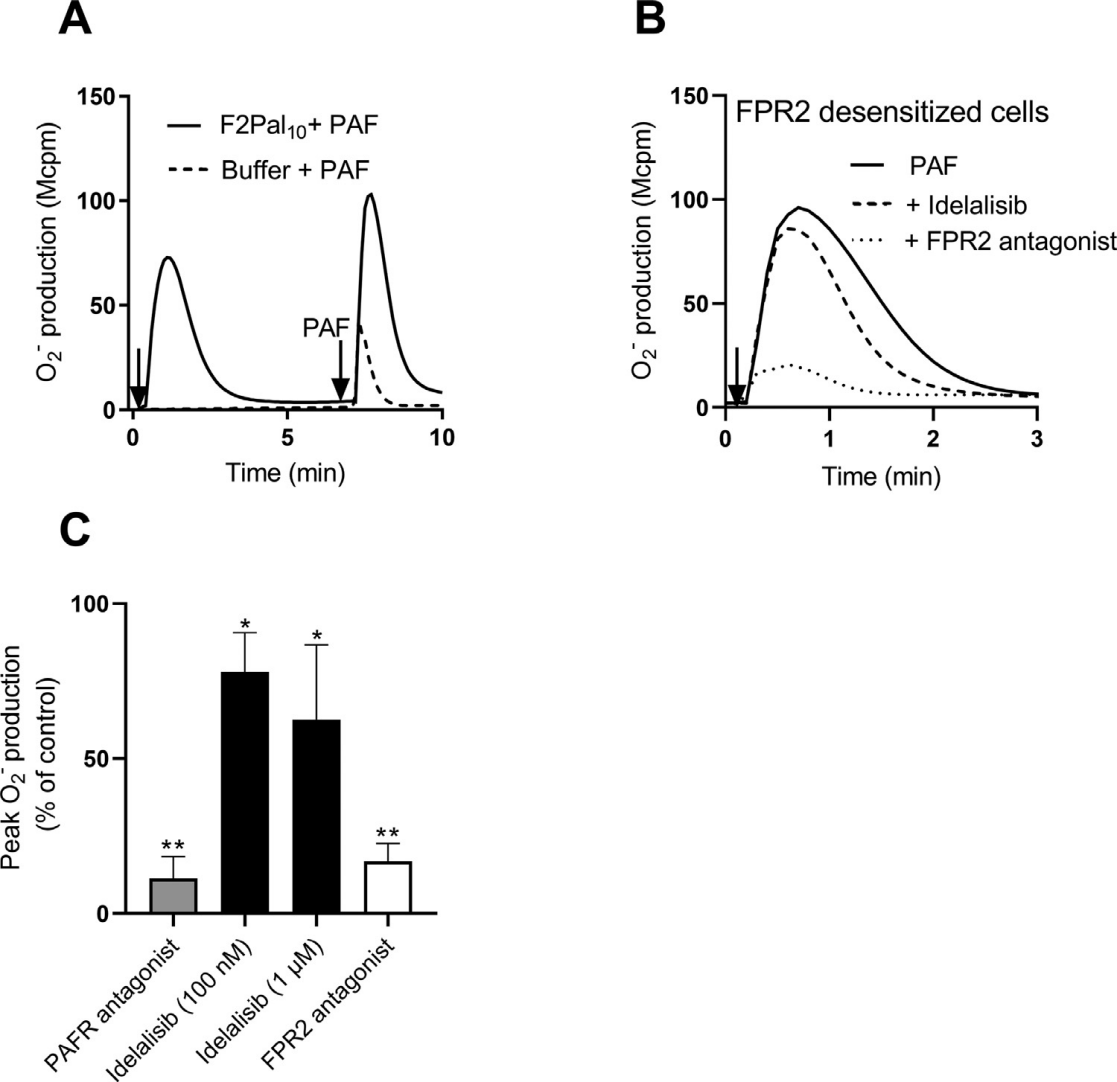
Effect of the PI3K-δ inhibitor Idelalisib on O_2_^−^ production induced upon FPR2 reactivation by PAF.

Data describes the effect of the PI3K-δ inhibitor Idelalisib on neutrophil superoxide production induced by F2Pal_10_ (agonist for FPR2), PAF (agonist for PAFR) and PMA (receptor-independent PKC activator). Neutrophil activation and ROS release induced by F2Pal_10_, PAF and PMA in the presence or absence of the PI3K-δ inhibitor are shown (Fig. 1). Also, data of the effect of the PI3K-δ inhibitor on PAF-induced reactivation of F2Pal_10_ desensitized neutrophils are provided; the effects of FPR2 and PAFR selective antagonists are included for comparison (Fig. 2).

Neutrophils (10^5^) were incubated for five min at 37 °C in the absence (agonist alone) or presence of the δ-isoform specific inhibitor Idelalisib (+ Idelalisib) before stimulation with PMA, F2Pal_10_ or PAF and measurement of O_2_^−^production with an isoluminol-enhanced chemiluminescence system. (**A-B)** Neutrophils incubated with or without Idelalisib (1 µM) were stimulated with PMA (50 nM). Data are presented as **A)** one representative chemiluminescence trace out of three and **B)** peak O_2_^−^ production compared with or without Idelalisib (1 µM) (mean + SD, *n* = 3). Statistical analysis was performed using paired Student's *t*-test to compare the inhibitor treated cells to control cells. **C-D)** Neutrophils incubated with or without different concentrations of Idelalisib (1 µM, 200 nM, 100 nM, 10 nM and 1 nM) were stimulated with F2Pal_10_ (500 nM). **E-F)** Neutrophils incubated with or without different concentrations of Idelalisib (1 µM, 100 nM, 10 nM, 5 nM and 1 nM) were stimulated with PAF (100 nM). **C, E)** Data are presented as one representative trace with or without Idelalisib (1 µM) *n* ≥ 4. **D** and **F)** Idelalisib dose-dependent inhibition (total O_2_^−^release, area under curve), as the normalized response in the absence of Idelalisib, and the fitted curve (mean ± SD *n* ≥ 4). The IC_50_ value and 95% of confidence interval (CI) are given.

**A)** Neutrophils (10^5^) were stimulated with the FPR2 agonist F2Pal_10_ (500 nM; solid line, first arrow) or buffer (dashed line). When the response had declined, the FPR2 desensitized cells were subsequently activated with a second stimulation with PAF (100 nM, indicated by the second arrow). **B**) F2Pal_10_ (500 nM) desensitized neutrophils incubated one min with Idelalisib (100 nM), FPR2 antagonist (CN6, 100 nM) or buffer before PAF stimulation (100 nM, indicated by the arrow). **A**-**B)** One representative O_2_^−^ trace out of six is shown. **C)** Quantification of inhibitory effects of Idelalisib (1 µM, 100 nM), FPR2 antagonist (CN6, 100 nM), and PAFR antagonist (WEB, 1 µM) on PAF-induced FPR2 reactivation. The inhibitors were added one min before stimulation of F2Pal_10_ (500 nM)-desensitized neutrophils with PAF (100 nM) and the peak O_2_^−^values for each condition were determined. The results are presented as the percent remaining PAF-induced NADPH-oxidase activity in FPR2-desensitized cells after treatment with the inhibitors as compared to FPR2-desensitized cells in the absence of inhibitors (control; mean + SD, *n* = 6). Statistical analysis was performed using one-way ANOVA followed by Dunnet's multiple comparison to control.

## 2 Experimental design, materials and methods

### 2.1 Neutrophil isolation

Human neutrophils were isolated from buffy coats as described [1], and diluted to 1 × 10^6^/mL in Krebs-Ringer phosphate buffer (KRG) containing glucose (10 mM), Mg^2+^ (1.5 mM), and Ca^2+^ (1 mM). The cells were kept on ice until use.

### 2.2 NADPH-oxidase activity

GPCR agonist-induced extracellular O_2_^−^ production was measured by an isoluminol-enhanced chemiluminescence system, described previously [2]. The O_2_^−^ production was measured in 6-channel Biolumat LB 9505 (Berthold Co, Wildbad, Germany), using disposable 4-mL polypropylene tubes with a 0.9 mL reaction mixture. Neutrophils (1 × 10^5^), HRP (4 U/mL), isoluminol (2 × 10^−5^ M) and optimal antagonist/inhibitor (KRG for control) were incubated (five min 37 °C) before stimulation with 0.1 mL agonist and the release of O_2_^−^was measured as light emission over time. FPR2 desensitized neutrophils were obtained by pre-activating neutrophils with the FPR2 specific agonist F2Pal_10_ [3], [4], [5]. When the response had returned to the baseline, these neutrophils were regarded as FPR2 desensitized (non-responsive to an additional of the same agonist or agonists that bind to the same receptor). For reactivation experiments, the FPR2 desensitized neutrophils were stimulated with PAF and the receptor specific antagonist or inhibitors were added one minute prior to the addition of PAF, as indicated in the figure legends. Some data (Fig. 1A, C, E and 2A-B) are shown with a representative chemiluminescence kinetics, abscissa time (min) and ordinate chemiluminescence arbitrary units (Mega counts per minute, Mcpm).

### 2.3 Chemicals

The FPR2 pepducin agonist F2Pal_10_ (Pal-KIHKKGMIKS, amino acid sequence from a part of third intracellular loop of FPR2), was from Caslo Laboratory (Lyngby, Denmark). The PI3K-δ specific inhibitor Idelalisib was purchased from Selleckchem (Zürich, Switzerland). PAF was obtained from Avanti Polar Lipids (Alabaster, AL, USA). The FPR2 peptidomimetic antagonist CN6 [6] was a kind gift provided by Henrik Franzyk (Copenhagen, Denmark). The PAFR antagonist WEB-2086 (WEB) was from Tocris Bioscience (Bristol, United Kingdom). Phorbol 12-myristate 13-acetate (PMA) and isoluminol were purchased from Sigma-Aldrich (St. Louis, MO, USA). Krebs-Ringer phosphate buffer supplemented with glucose (10 mM), Ca^2^^+^ (1 mM) and Mg^2+^ (1.5 mM) (KRG; pH 7.3) was made in house. Dextran and Ficoll-Paque were obtained from GE Healthcare Life Sciences (Uppsala, Sweden). Horseradish peroxidase (HRP) was obtained from Boehringer Mannheim (Germany).

### 2.4 Data analysis

Data analysis was performed with Graph Pad Prism version 8.0a (GraphPad Software, La Jolla, CA, USA). Data were analyzed with a paired Student´s *t*-test or a one-way ANOVA followed by Dunnet's multiple comparison test; details are stated in the respective figure legends. Statistical significant differences are indicated by *p ≤ 0.05. **p ≤ 0.01. Each independent experiment was performed as biological replicates with neutrophils isolated from individual blood donors.

## Ethics statement

Buffy coat blood samples were obtained from healthy adults from the blood bank at Sahlgrenska University Hospital. Ethics approval was not needed since the buffy coats were provided anonymously and could not be traced back to a specific individual. This is in line with Swedish legislation section code 4§ 3p SFS 2003:460 (Lag om etikprövning av forskning som avser människor).

## Declaration of Competing Interest

The authors declare that they have no known competing financial interests or personal relationships which have, or could be perceived to have, influenced the work reported in this article.
